# Gut–Joint Axis: The Role of Physical Exercise on Gut Microbiota Modulation in Older People with Osteoarthritis

**DOI:** 10.3390/nu12020574

**Published:** 2020-02-22

**Authors:** Alessandro de Sire, Roberto de Sire, Valentina Petito, Letizia Masi, Carlo Cisari, Antonio Gasbarrini, Franco Scaldaferri, Marco Invernizzi

**Affiliations:** 1 Physical and Rehabilitation Medicine, Department of Health Sciences, University of Eastern Piedmont “A. Avogadro”, 28100 Novara, Italy; cisari50@gmail.com; 2 Rehabilitation Unit, “Mons. L. Novarese” Hospital, Moncrivello, 13040 Vercelli, Italy; 3 Gastroenterology Unit, Department of Clinical Medicine and Surgery, School of Medicine “Federico II”, 80131 Naples, Italy; 4 Istituto di Patologia Speciale Medica, Università Cattolica del Sacro Cuore, 00168 Rome, Italy; valentina.petito@unicatt.it (V.P.); letizia.masi94@gmail.com (L.M.); antonio.gasbarrini@unicatt.it (A.G.); 5 UOC Medicina Interna e Gastroenterologia, Area Gastroenterologia e Oncologia Medica, Dipartimento di Scienze Gastroenterologiche, Endocrino-Metaboliche e Nefro-Urologiche, Fondazione Policlinico Universitario A. Gemelli IRCCS, 00168 Rome, Italy; 6 Physical Medicine and Rehabilitation Unit, University Hospital “Maggiore della Carità”, 28100 Novara, Italy

**Keywords:** gut microbiota, osteoarthritis, knee osteoarthritis, exercise, rehabilitation, gastrointestinal microbiome, inflammaging, gut dysbiosis, diet, probiotics

## Abstract

Osteoarthritis (OA) is considered one of the most common joint disorders worldwide and its prevalence is constantly increasing due to the global longevity and changes in eating habits and lifestyle. In this context, the role of gut microbiota (GM) in the pathogenesis of OA is still unclear. Perturbation of GM biodiversity and function, defined as ‘gut dysbiosis’, might be involved in the development of inflammaging, one of the main risk factors of OA development. It is well known that physical exercise could play a key role in the prevention and treatment of several chronic diseases including OA, and it is recommended by several guidelines as a first line intervention. Several studies have shown that physical exercise could modulate GM composition, boosting intestinal mucosal immunity, increasing the Bacteroidetes–Firmicutes ratio, modifying the bile acid profile, and improving the production of short chain fatty acids. Moreover, it has been shown that low intensity exercise might reduce the risk of gastrointestinal diseases, confirming the hypothesis of a strict correlation between skeletal muscle and GM. However, up to date, there is still a lack of clinical trials focusing on this research field. Therefore, in this narrative, we aimed to summarize the state-of-the-art of the literature regarding the correlation between these conditions, supporting the hypothesis of a ‘gut–joint axis’ and highlighting the role of physical exercise combined with adequate diet and probiotic supplements in rebalancing microbial dysbiosis.

## 1. Introduction

Osteoarthritis (OA) is the most common musculoskeletal disease, characterized by progressive articular cartilage loss, formation of osteophytes, subchondral bone remodeling, and chronic joint inflammation [1]. OA mainly targets the hip and knee joints, although other skeletal sites (i.e., hands, feet, spine) might be affected by this condition [2]. In particular, knee OA has a prevalence of 3.8% and an incidence of 12% in the elderly population and occurs with joint pain and peri-articular muscle weakness with a subsequent loss of function, increased disability, and reduction of health-related quality of life [3,4]. Indeed, an early diagnosis of OA is crucial to set up a prompt, effective, and patient-oriented treatment in order to prevent the age-related degenerative progression of this pathological condition [5]. The etiology of OA still remains unknown, but several risk factors have been identified such as age, sex, obesity, diet, physical inactivity, abnormal loading of the joints, metabolic syndrome, and inflammaging [6]. These two latter conditions are key components of OA onset and evolution, and recently the microbial dysbiosis induced by quantitative and qualitative alterations of the gut microbiota (GM) has been shown to sustain a chronic systemic low-grade inflammation, boosting the main pathophysiological mechanisms underpinning OA [7].

The GM colonizes the entire gastrointestinal tract in a variable way and represents a real ecosystem, weighing about 1.5 kg, and is composed of more than 10^14^ bacteria and more than 1000 species as well as fungi, viruses, phages, parasites, and archea [8]. The most representative bacterial phyla of healthy GM are Bacteroidetes and Firmicutes, followed by Actinobacteria, Fusobacteria, and Proteobacteria, whereas the most representative genera are Bacteroides, Faecalibacterium, and Bifidobacterium [9]. GM has several functions such as nutrient absorption, maintenance of metabolic homeostasis, protection from infections, and development of systemic and mucosal immunity [10,11]. Modifications in the GM composition and metabolic activity might alter the immune response and host metabolism, leading to a constant low-grade inflammation status with consequent development of musculoskeletal impairment and frailty [12,13]. Furthermore, an expert consensus of the European Society for Clinical and Economic Aspects of Osteoporosis, Osteoarthritis and Musculoskeletal Diseases has recently hypothesized that GM is a major hidden risk factor for OA [7].

Several studies [14,15,16] have hypothesized the linkage between GM and osteoarthritis, taking into account the potential correlations between serum levels of bacterial metabolites and joint degeneration, however, at present, no systematic reviews have clearly described this topic.

Moreover, it has been recently hypothesized that physical exercise could also play a key role in modifying GM composition [17] and at the same time, it could reduce the risk of gastrointestinal diseases [18] such as inflammatory bowel disease, considering the emergent well-studied relationship between muscle function and GM [13,19,20,21].

In this narrative review, we aimed to show the state-of-the-art about the previously hypothesized correlations between GM alterations and OA in order to highlight new potential therapeutic directions and types of intervention in the ‘gut–joint axis’ modulation.

## 2. Inflammaging

The association between OA and low-grade systemic and local inflammation is well known [22,23]; indeed, OA etiopathogenic mechanisms might include epigenetic alterations, mitochondrial dysfunction, cellular senescence, and an age-related increased production of proinflammatory mediators, resulting in a pro-inflammatory state, referred to as ‘inflammaging’ [24,25].

The presence of physiological TGF-β signaling is mandatory for the maintenance of correct architecture and functioning of the articular cartilage [26]. The binding of TGF-β to its type II receptor leads to the recruitment and phosphorylation of a type I receptor. In more detail, TGF-β could signal both via the canonical type I receptor ALK5 (TGFBR1) or via the ALK1 (ACVRL1) receptor in chondrocytes [27,28], and a positive correlation between the ALK1 expression levels and MMP13 expression in human OA cartilage has been previously demonstrated [27]. Therefore, an adequate balance between ALK5 and ALK1 is mandatory to maintain cartilage homeostasis and the loss of ALK5 signaling could result in the loss of cartilage integrity [29]. Indeed, the ALK1/ALK5 ratio was demonstrated to be significantly increased in old C57Bl/6 mice compared to the young ones, leading TGF-β signaling via the Smad1/5/8 route and chondrocyte differentiation into cells with an autolytic phenotype [27]. Furthermore, the chronic systemic inflammation in the geriatric population is sustained by pro-inflammatory cytokines such as interleukin-6 (IL-6) and tumor necrosis factor-alpha (TNF-a), whose serum levels commonly increase with age and are strongly correlated with the risk of OA progression [30,31].

A direct correlation between aging and gut dysbiosis has been previously demonstrated and is mainly characterized by an increase of pro-inflammatory anaerobes species and a consistent decrease in anti-inflammatory species such as *Faecalibacterium prauznitzii* [32]. As evidence of these hypotheses, it was later shown how longevity was inversely correlated with the alpha diversity of GM [33,34]. Finally, a typical feature of the aging process is the so-called “leaky gut syndrome”, an increased intestinal permeability leading to the translocation of microbial products through the intestinal tight junctions, resulting in endotoxemia and consequently in a pro-inflammatory status [35].

A possible link between gut dysbiosis and OA might be suggested by the correlation between the serum levels of bacterial metabolites and joint degeneration [16]. This correlation has been described in rodent models and humans affected by OA and is characterized by increased levels of circulatory inflammatory markers including bacterially produced lipopolysaccharides (LPS) [36,37], suggesting that microbiome-derived proinflammatory metabolites could play a putative role in OA pathogenesis. Moreover, it has recently been shown that the subchondral dysbiosis resulting from the bacterial translocation from GM to the joint, might support the development of OA [14]. Therefore, considering gut dysbiosis and the leaky gut syndrome as cofactors in the development of inflammaging and consequently of OA [38], it is likely to hypothesize the existence of a ‘gut–joint axis’ [7,14,15,16].

## 3. Obesity and Metabolic Syndrome

Diet is one of the major environmental factors contributing to the inter-individual differences in physiologic GM microbiota composition [39,40]. Nutrients are fundamental not only for human health, but also for the well-being of the trillions of microbes that compose the GM [41]. It has been demonstrated that a high-fat diet was associated with a reduction of *Bacteroidetes* and an overgrowth of opportunistic pathogens, promoting leaky gut syndrome, and enhancing systemic inflammation and insulin resistance [42]. In contrast, the production of short-chain fatty acids (SCFAs) by healthy gut bacteria such as Faecalibacterium, Succinivibrio, and Butyricimonas are able to induce an increase of glucose uptake and insulin sensitivity [43]. Similarly, mice models have shown that SCFAs might have a key role in maintaining bone homeostasis, downregulating the pro-inflammatory stimuli sustained by regulatory T-cells and inhibiting bone resorption through a direct inhibition of osteoclast activity [44].

It is important to underline the role of an adequate diet and the use of dietary supplements in the ‘active aging’ population [45,46]. Indeed, these two interventions combined with vitamin D and amino acid supplementation and physical exercise could prevent several sequelae of the aging process including sarcopenia, as shown by several studies in the literature [47,48,49,50,51,52,53]. Finally, it has been demonstrated that there is a strong correlation between the adherence to a Mediterranean diet and a lower risk of OA manifestation, mediated by the well-known anti-inflammatory activity of fibers [54]. Taken together, these findings suggest that a rich in fiber nutritional approach like the Mediterranean diet combined with an adequate intake of micronutrients and physical exercise might be a cornerstone to maintain good health status, particularly in older people.

The excess of adipose tissue is the result of a multifactorial process including genetic and environmental factors with an imbalance between energy intake and expenditure, which might lead to an increased risk of developing metabolic syndrome, characterized by hyperglycemia, hypertriglyceridemia, dyslipidemia, and hypertension [55]. Recent studies have focused on the association between body mass index (BMI) and GM composition [56,57]. In particular, Gao et al. [56] investigated the fecal microbiome composition, profiled via 16S rRNA gene sequencing, on 551 participants categorized as underweight, normal, overweight, and obese, based on their BMI. The authors showed that the bacterial community of the underweight group had a significantly higher alpha diversity than other groups and, if stratified by gender, the alpha diversity across the BMI was maintained in females. Moreover, an enrichment of Fusobacteria was observed in the fecal microbiota of obese males and an abundance of Actinobacteria in obese females. Finally, the butyrate-acetoacetate CoA-transferase was enriched in obese subjects, resulting in an excess of energy accumulation. These findings are in line with previous data in the literature showing a correlation between the development of obesity-associated OA and gut dysbiosis [58,59] in animal models, acting through an interaction with the innate immune system at both systemic and local levels [59]. Lipotoxicity is a typical feature of the metabolic syndrome that might promote the production of proinflammatory cytokines with the consequent recruitment of mast cells, macrophages, and dendritic cells in the adipose tissue, inducing a chronic low-grade inflammatory status [60].

Thus, considering these proinflammatory effects, obesity is considered one of the main risk factors of OA [60], and it seems to also negatively influence muscle mass and function in older people [61,62]. In animal models, a high-fat/high-sucrose diet is able to promote knee joint damage, with an increase of the intestinal permeability and a consequent increase of serum levels of LPS, supporting the hypothesis of a linkage between gut dysbiosis, metabolic inflammation, and OA [7].

## 4. Gut Microbiota Modulation as Treatment of Osteoarthritis

The rebalancing of GM could have positive effects on the treatment of dysbiosis-related diseases; in particular, GM modulation might be obtained through specific diets, probiotics (live organisms), and prebiotics (indigestible and non-absorbable substances) supplementation [63,64,65]. Probiotics such as Bifidobacterium and Lactobacillus exert their role by promoting gut health through the regulation of pH levels and the colonization resistance [63,64,65]. On the other hand, prebiotics are able to stimulate the activity and growth of several bacteria in the gastrointestinal tract, promoting the fermenting process involved in the generation of SCFAs, which are considered key regulatory metabolites of gut wellness [63,64,65].

In this context, it was hypothesized that GM modulation is able to modify the onset and the progression of OA. Previous studies have shown that the administration of probiotics significantly decreased the expression of pro-inflammatory cytokines in rat knee cartilage during monoiodoacetate-induced OA [66]. Korotkyi et al. described the beneficial effects of a probiotic diet on the expression of prostaglandin endoperoxide synthase-2 (PTGS2) and transforming growth factor beta-1 (TGFb1), suggesting a potential perspective to improve the standard treatment of OA [66]. Furthermore, prebiotic supplementation in combination with aerobic exercise could prevent knee joint damage, otherwise observed in an obesity-induced OA rat model [67]; in this study, the authors highlighted that the integrity of the knee joint obtained in the prebiotic diet and physical activity group was associated with the maintenance of GM homeostasis and the prevention of endotoxemia.

The main linking factor between GM and OA seems to be represented by the common chronic low-grade inflammation, supporting a new OA phenotype, indicated as the ‘metabolic OA’ [15]. However, it has been hypothesized that the microbial community shifts induced by antibiotics, a germ-free environment, or high-fat might have a key role in the development of OA, suggesting that active microbiome modulation could be an effective therapeutic option to counter joint degeneration in OA [16]. Finally, it has been suggested that dietary supplements and nutraceuticals might exert their positive effects on health status in the elderly [45] through quantitative and qualitative modifications of the GM [16].

Although previous studies have investigated the gut–joint axis in animal models, clinical studies in humans are lacking and future perspectives should focus on a more detailed understanding of the in vivo effects of probiotic and/or prebiotic administration in OA patients.

## 5. Physical Exercise and Gut Microbiota

**Figure 1:**
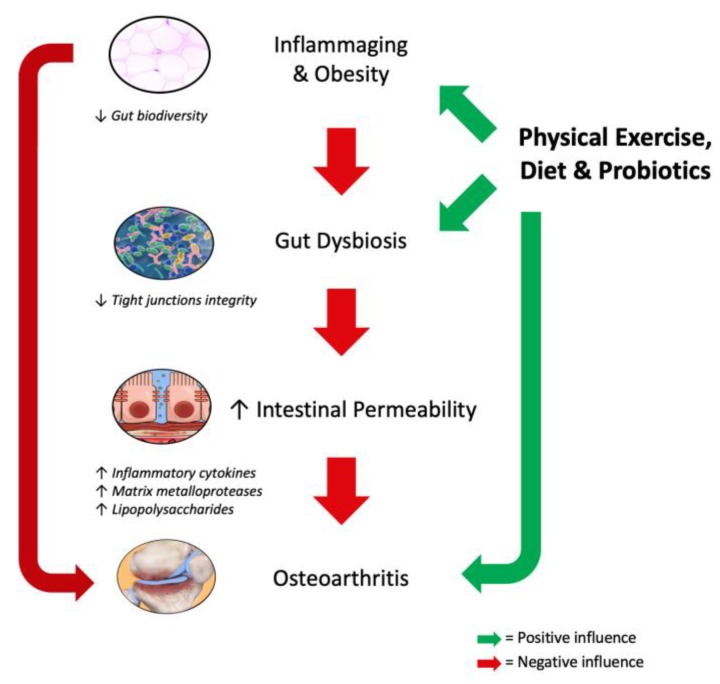
Pathogenic mechanisms of the ‘gut–joint axis’ and the potential role of physical exercise combined with diet and probiotics.

Physical exercise plays a fundamental role in the prevention and treatment of several chronic diseases including OA [68]; and it is recommended by several guidelines as a first line intervention in the management of this condition [69,70,71]. Pathogenic mechanisms of the ‘gut–joint axis’ and the potential therapeutic role of physical exercise combined with diet and probiotics are depicted by Figure 1.

Recently, several studies have shown that physical exercise could modulate GM composition [17,20,21], boost intestinal mucosal immunity [72], increase the Bacteroidetes–Firmicutes ratio [73,74], modify the bile acid profile [75], and improve the production of short chain fatty acids such as n-butyrate, acetate, and propionate [73,76]. In more detail, it has to be noted that acetate and propionate are carried in the bloodstream to several organs as substrates for local energy metabolism (i.e., gluconeogenesis) [77].

It has been previously shown that low intensity exercise might reduce the risk of gastrointestinal diseases (i.e., colon cancer, diverticulosis, and inflammatory bowel disease), taking into account the strict correlation between skeletal muscle and GM [13,17,18,19,20,21]. In more detail, physical activity might modify the transient stool time and the contact time between the pathogens and the gastrointestinal mucus layer, resulting in the gastrointestinal tract modifications above-mentioned [78].

How can the GM be modified in patients undergoing intense physical activity? At present, the question remains unanswered. However, it has been demonstrated that GM can control oxidative stress and inflammatory responses, improving metabolism and energy expenditure [75]. It is important to underline that this is a two-way relationship, as shown by the key role of exercise in reducing inflammatory infiltrate and protecting the morphology and integrity of the intestinal tract, independently from the diet [79].

Does exercise have a role in contrasting inflammaging independently of an adequate diet? Evans et al. showed that physical activity seems to prevent diet-induced obesity through a GM modulation that results in a microbial composition similar to lean mice [73]. Moreover, it was recently demonstrated that cardiorespiratory fitness, independent from diet, was correlated with increased GM diversity in healthy humans [80]. At the same time, exercise might prevent obesity, also changing the percentage of major bacterial phyla in obese-induced mice through high fat feeding, as shown by the inverse correlation between the Bacteroidetes–Firmicutes ratio and the total distance run [73].

All these data were confirmed by a recent study performed by Campbell et al. that demonstrated the presence of bacteria related to *Faecalibacterium prausnitzii* in mice undergoing a physical exercise protocol [79]. These bacteria commonly protect the digestive tract through butyrate production and lowering the oxygen tension in the lumen, boosting a flavin/thiol electron shuttle. Furthermore, it has been demonstrated that fit individuals showed a gut microbial profile enriched in Clostridiales, Roseburia, Lachnospiraceae, and Erysipelotrichaceae bacteria, which are all butyrate-producers and considered as indicators of gut health [80].

However, up to now, despite these evidences, the conundrum between physical exercise and probiotic interventions in the modulation of GM is far from being understood in detail. Moreover, few studies including the ones previously cited, took into account specific parameters of physical function. Román et al. [81] have recently shown in a randomized trial that the administration of a probiotic blend to cirrhotic patients could enhance the physical performance, according to the timed up and go test (TUG); indeed, patients treated with probiotics showed an improvement in TUG (*p* = 0.015) and gait speed (*p* = 0.02), and a trend toward a lower incidence of falls at the follow-up (*p* = 0.10). Table 1 describes all the studies that have investigated the correlation between physical exercise and GM.

## 6. Conclusions

This narrative review showed how several studies supported the hypothesis of a ‘gut–joint axis’ and in this context, physical exercise interventions combined with different nutritional approaches might play a key role in rebalancing microbial dysbiosis and could be considered as adjuvant treatments in dysbiosis-associated diseases.

However, these findings remain anecdotal and data on quantitative and qualitative microbiome composition in OA patients are still lacking and more research efforts are needed to understand the shared pathways and synergies between exercise, diet, and probiotics in GM modulation. Thus, to address these issues, future research should focus on several aspects: (1) a detailed assessment of the correlation between GM, prebiotics/probiotics, and nutraceuticals to create effective therapeutic interventions to counteract OA pathogenesis and progression; (2) investigate new potential biomarkers related to inflammaging and gut dysbiosis (i.e., inflammatory cytokines, matrix metalloproteases, and LPS) that are able to predict OA onset and monitor therapeutic intervention efficacy; and (3) to investigate how new cutting edge GM therapeutic interventions might impact host biology and might protect from OA onset.

## Figures and Tables

**Table 1:** Main studies included in the present narrative review that have investigated the correlation between physical exercise and gut microbiota (GM).

Authors, Year	Study Design	Population	Intervention/Groups	Outcomes	Main Results	Conclusions
Bermon S, et al. 2015 [75]	Review	Animals/Humans	N/A	N/A	N/A	Exercise might modulate and help to restore the GM when altered by a high fat diet.
Campbell SC, et al. 2016 [76]	Randomized controlled trial	Thirty-six, 6-week old C57BL/6NTac male mice	The mice were divided into our groups: 1) lean sedentary (LS); 2) diet-induced obesity (DIO) sedentary (OS); 3) lean exercise (LX); 4) DIO exercise (OX) groups.	Duodenum/ileum tissues were fixed for immune-histo-chemistry for occludin, Ecadherin, and cyclooxygenase-2 (COX-2). Bacterial communities were assayed in fecal samples using terminal restriction fragment length polymorphism (TRFLP) analysis and pyrosequencing of 16S rRNA gene amplicons.	LS mice presented normal histologic villi while OS mice had similar villi height with more than twice the width of the LS animals. Both LX and OX mice duodenum and ileum were histologically normal. COX-2 expression was the greatest in the OS group, followed by LS, LX and OX. The TRFLP and pyrosequencing indicated that members of the Clostridiales order were predominant in all diet groups. Specific phylotypes were observed with exercise, including Faecalibacterium prausnitzi, Clostridium spp., and Allobaculum spp.	Exercise has a strong influence on gut integrity and host microbiome.
Cerda B, et al. 2016 [72]	Review	Animals/Humans	N/A	N/A	N/A	The release of hormones that occurs during exercise could modify the GM profile of subjects performing physical exercise at certain intensities or durations.
Estaki M, et al. 2016 [80]	Cross-sectional study	Healthy young adults aged 18–35 years	A continuous incremental ramp maximal exercise test on an electronically braked cycle ergometer to assess the Peak oxygen uptake (VO_2_ peak). According to the VO_2_ peak, participants were categorized into 3 groups: 1) low (LOW); 2) average (AVG); 3) high fitness (HI).	Peak oxygen uptake (VO2peak), as an indicator of physical fitness. Short-chain fatty acids (acetic, propionic, heptanoic, valeric, caproic, and butyric acid) were analyzed from the feces by gas chromatography (GC) as described previously Using high-throughput sequencing to analyze fecal microbiota		GM diversity in healthy humans is associated with aerobic fitness. Moreover, the GM profile of fit individuals appears to favor butyrate production (indicator of gut health) through increases in Clostridiales, Roseburia, Lachnospiraceae, and Erysipelotrichaceae genera.
Evans CC, et al. 2014 [73]	Randomized controlled trial	Male C57BL/6 littermate mice (5 weeks)	Mice were divided into 4 groups: 1) low fat and sedentary (LF/Sed); 2) low fat and exercise (LF/Ex); 3) high fat and sedentary (HF/Sed); 4) high fat and exercise (HF/Ex). LF/Ex and HF/Ex cages were equipped with a wheel and odometer to record Ex.	Fecal samples were collected at baseline, 6 weeks and 12 weeks and used for bacterial DNA isolation. DNA was subjected both to quantitative PCR using primers specific to the 16S rRNA encoding genes for Bacteroidetes and Firmicutes and to sequencing for lower taxonomic identification using the Illumina MiSeq platform	HF diet resulted in significantly greater body weight and adiposity as well as decreased glucose tolerance that were prevented by voluntary exercise (*p* < 0.05). Sequencing demonstrated exercise-induced changes in the percentage of major bacterial phyla at 12 weeks. A correlation between total Ex distance and the ΔCt Bacteroidetes/ΔCt Firmicutes ratio from qPCR demonstrated a significant inverse correlation (*r*^2^ = 0.35, *p* = 0.043).	The exercise induces a unique shift in the GM that is different from dietary effects. The Bacteroidetes phylum increased while it decreased firmicutes in a manner that was proportional to the distance run in mice fed with high-fat diet. GM changes might play a role in exercise prevention of the high-fat diet-induced obesity.
Hsu YJ, et al. 2015 [76]	Prospective study	Male mice aged 12 weeks	The mice were divided into 3 groups: 1) specific pathogen-free (SPF); 2) germ-free (GF) 3) Bacteroides fragilis (BF) gnotobiotic mice Swimming was performed in plastic containers.	Endurance Swimming, weight of liver, muscle, brown adipose, and epididymal fat pads.	Endurance swimming time was longer for SPF and BF than GF mice, and the weight of liver, muscle, brown adipose, and epididymal fat pads was higher for SPF and BF than GF mice.	GM status could be crucial for exercise performance and its potential action linked with the antioxidant enzyme system in athletes.
Mach N & Fuster-Botella D. 2017 [75]	Review	Animals/Humans	N/A	N/A	N/A	Exercise might modulate and help to restore the GM when altered by a high fat diet.
Monda V, et al., 2017 [17]	Review	Animals/Humans	N/A	N/A	N/A	Exercise seems to be an environmental factor that might determine changes in the GM composition with possible benefits for the host. Physical exercise is able to enrich the microflora diversity and to improve the Bacteroidetes–Firmicutes ratio, that might contribute to reduce obesity-associated pathologies, and gastrointestinal disorders
Morita E, et al. 2019 [74]	Prospective study	Thirty-two sedentary healthy elderly women	The subjects were allocated into two groups receiving different exercise interventions, trunk muscle training or aerobic exercise training.	The GM composition in fecal samples was determined before and after the training period. Clinical outcome measures were: a) daily physical activity, using an accelerometer; b) trunk muscle strength, by the modified Kraus–Weber test; c) cardiorespiratory fitness, through 6-min walk test (6MWT).	Exercise intervention modified microbiota composition and improved 6MWT.	Aerobic exercise targeting an increase of the time spent in brisk walking could increase intestinal Bacteroides correlated with an improved cardiorespiratory fitness in a cohort of healthy elderly women.
Picca A, et al. 2020 [21]	Cross-sectional study	Thirty-five participants community-dwellers aged more than 70 years.	The presence of PF&S was established according to physical frailty, based on: a) Short Physical Performance Battery between 3 and 9; b) low appendicular muscle mass; c) absence of mobility disability. They were divided into 2 groups: 18 older adults with physical frailty and sarcopenia (PF & S), mean aged 75.5 ± 3.9 years, and 17 non-PF & S controls, mean aged 73.9 ± 3.2 years	A multi-marker immunoassay was used to measure circulating levels of a panel of inflammatory markers. Serum concentrations of 37 amino acids and derivatives were determined by ultraperformance liquid chromatography/ mass spectrometry. Total genome DNA was extracted from fecal samples using the QIAmp Fast DNA Stool mini kit (Qiagen, Germany).	PF&S participants showed higher serum concentrations of aspartic acid, lower circulating levels of concentrations of threonine and macrophage inflammatory protein 1, increased abundance of Oscillospira and Ruminococcus microbial taxa, and decreased abundance of Barnesiellaceae and Christensenellaceae.	The Authors showed a specific relationship among GM, systemic inflammatory mediators, and metabolic alterations in older adults with PF&S.
Ticinesi A, et al. 2019 [20]	Review	Animals/Humans	N/A	N/A	N/A	The presence of a gut-muscle axis involved in the pathophysiology of PF & S is biologically plausible and is supported by a limited number of animal and human studies. However, the Authors conclude affirming that the causal link was still uncertain.
